# Research on overburden structural characteristics and support adaptability in cooperative mining of sectional coal pillar and bottom coal seam

**DOI:** 10.1038/s41598-024-62375-7

**Published:** 2024-05-20

**Authors:** Du Feng, Fan Xuan, Li Zhenhua, Cao Zhengzheng, Wang Wenqiang, Lu Feifei, Jiang Yufei

**Affiliations:** 1 School of Energy Science and Engineering, Henan Polytechnic University, Jiaozuo, 454000 Henan China; 2 Henan Mine Water Disaster Prevention and Control and Water Resources Utilization Engineering Technology Research Center, Henan Polytechnic University, Jiaozuo, 454000 Henan China; 3 Collaborative Innovation Center of Coal Work Safety and Clean High Efficiency Utilization, Jiaozuo, 454000 Henan China; 4 International Joint Research Laboratory of Henan Province for Underground Space Development and Disaster Prevention, Henan Polytechnic University, Jiaozuo, 454000 Henan China

**Keywords:** Cooperative mining, Overburden structure, Hydraulic support, Sectional coal pillar, Adaptability evaluation, Coal, Civil engineering

## Abstract

In the mining process of the II1 coal seam at Zhaogu No. 2 coal mine, a method of stratified mining is employed, leaving relatively wide coal pillars in sections. To enhance the resource recovery rate, the mine carries out the cooperative mining of the sectional coal pillars and the lower layer coal seam. The 14,022 cooperative working face of fully-mechanized and fully-mechanized top-coal caving at Zhaogu No. 2 coal mine is taken as the research object. Through numerical simulation, theoretical calculations, and on-site industrial trials, a comprehensive analysis of the overburden structural characteristics and the support adaptability at the working face is conducted. It is clarified that a stress arch bearing structure can be formed above the sectional coal pillars during cooperative mining, and this structure is controlled by key strata. The formation of a stress arch bearing structure in the overburden above the sectional coal pillars provides protection for the underlying mining area. A formula for calculating the working resistance of hydraulic supports under the stress arch in sectional coal pillar is derived. Based on these results, the working resistance of hydraulic supports in the coal pillar area is calculated and selected. Field application shows that the working resistance of the support is 10,000 kN in the fully-mechanized top-coal caving working face, and is 9000 kN in fully-mechanized working face, meeting the support requirements and ensuring safe mining at the working face. This study provides a valuable engineering reference for achieving cooperative mining of abandoned sectional coal pillars and lower layer coal seam in stratified mining method.

## Introduction

Restricted by equipment technology and management level, some mining areas in Henan Province adopt layered mining in the process of mining thick coal seams and thus leave a large number of section coal pillars, resulting in waste of coal resources^1–3^. In order to improve the recovery rate of mine resources and prolong the service life of the mine, Zhaogu No. 2 coal mine jointly arranges the section coal pillar and the lower layer to form a fully mechanized mining face^4,5^. On the premise of ensuring safety, the recovery of coal resources in section coal pillars is helpful to improve the mining technology level of residual coal resources in China, realize the safe and efficient mining of mines, and is of great significance to alleviate the tense situation of energy demand in China^6,7^.

Li Shugang et al.^8^ studied the fracture evolution law of mining overburden compaction zone under different mining height conditions, and found that the increase of mining height will lead to the increase of compaction zone height. Wang Zhaohui et al.^9^ proposed that when the support stiffness is maintained at a certain value, the dynamic load impact of the roof can be alleviated, so that the support can yield pressure in time, thus ensuring the stability of the coal wall in the stope. Zhu Ye et al.^10^ clarified that different coal pillar size conditions are affected by the mining stress field, and the stress environment of the floor is different. Behera et al.^11^ analyzed the stability and spalling phenomenon of the working face based on the field conditions of the longwall working face in Godavari Valley coalfield, and clarified that the failure and collapse characteristics of the key roof were closely related to the instability of the working face. Wang et al.^12^ carried out research and analysis on the mining process of ultra-thick coal seam in 8.8 m large mining height working face by means of similar simulation, numerical simulation and field measurement, and clarified the movement and stress evolution law of overlying strata in working face. Peter et al.^13^ studied conventional gas wells penetrating through longwall chain pillars on the basis of an engineering example, and the results showed that the numerical simulation could accurately reflect the deformation of overlaying strata. Based on the S–R theory of the key rock blocks of the masonry beam structure, Han^14^ established the S–R stability criterion model of the masonry beam structure in the overlying rock of the long-wall goaf under the influence of the surface load, and deduced the slip and rotary instability discriminant formula. TMK A et al.^15^ studied the stability of a non-typical bleeder entry system at a U.S. longwall mine, showed very good correlation between modeled and expected gateroad loading during panel extraction. Feng et al.^16^ based on the analysis of the mechanical mechanism of thick key strata, determined the working resistance of the working face support under the goaf of shallow coal seams with close distance. Taking an underground coal mine in western Pennsylvania as an example, Sherizadeh et al.^17^ discussed the root cause of roof caving in this coal mine. It is clear that the bedding plane plays an important role in the geomechanical behavior of the underground excavation roof. The three-dimensional discrete element analysis method is used to evaluate the influence of the parameters such as the change of the strength characteristics of the direct roof rock mass, the change of the mechanical properties of the structural plane, the direction and size of the rock mass. Xu Jialin et al.^18^ based on the field measurement, simulation test and theoretical analysis of the first 7.0 m support fully mechanized mining face in China, proposed a method to determine the working resistance of the support when the key strata structure of different overburden strata in the fully mechanized mining face with large mining height is different. Islavath^19^ evaluated the stability of longwall working face from two aspects of displacement and stress by establishing a model combining working face and hydraulic support. Li Zhihua et al^20^ used theoretical analysis and numerical simulation to compare the mechanisms leading to instability and disaster in the overlying rock structures of general and thin-bedrock working faces, establishing a "macro-major-minor" structural mechanics model for mining overlying rock and determining the critical working resistance of supports under different overlying rock structures. Gonzalez-Nicieza^21^ used FLAC to model the working behavior to determine the maximum pressure that the footers on the coal can withstand, as well as the density of the struts and the conditions of the struts so that no penetration of the struts would be created. Yan Shaohong et al.^22^ addressed issues such as strong manifestations of mining pressure and insufficient basis for determining support resistance in high mining height faces, proposing the likelihood of forming a "short cantilever beam-hinged rock beam" structure and a formula for calculating the working resistance of supports in high mining height faces. Ju Jinfeng et al.^23,24^ summarized the structural forms of key layers in the overlying strata of extra-high mining faces in the Shendong mining area and their effects on the manifestation of mining pressure. Das et al.^25^ made it clear that the strength of the inclined coal pillar decreases with the increase of the inclination of the coal pillar through the strength formula derived. Verma et al.^26^ used finite element software to analyze the stress distribution at the convergence of hydraulic legs, abutments and roofs of hydraulic supports during longwall mining. Petr et al.^27^ used numerical simulation software to simulate and compare the yield volume of rock mass before and after stress reduction blasting technology to evaluate the buffer of additional stress transferred from the mining area to the safety pillar.

The existing results provide a reference for the safe mining of the working face, but there are few studies on different mining techniques for different mining heights in the same working face^28^. Therefore, theoretical calculation, numerical simulation and field industrial test are used to comprehensively analyze the movement law of overlying strata and the adaptability of support in working face, and the feasibility of hydraulic support is studied. This study can provide a good engineering reference for the realization of the co-mining of the residual section coal pillar and the lower layer in the layered mining.

## Project overview

The purpose of mixed mining is to give full play to the advantages of equipment and management, and to realize the one-time mining of coal pillars left in the upper layer and coal resources in the lower layer in the process of layered mining. In the same working face, the fully mechanized caving section and the fully mechanized mining section are mixed, and the fully mechanized caving support and the fully mechanized mining support are arranged jointly, and the coal mining and caving operations are coordinated according to a certain process. The fully mechanized caving section and the fully mechanized section of mixed mining have the characteristics of different coal thickness and different equipment on both sides.

**Figure 1:**
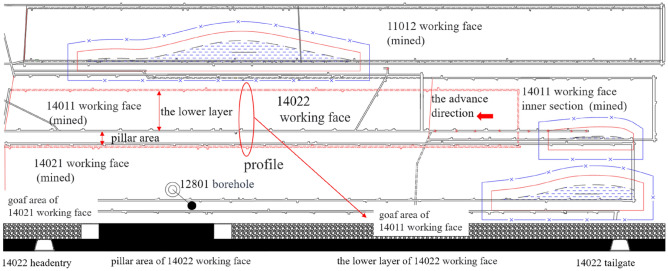
14,022 working face plan and profile map.

**Figure 2:**
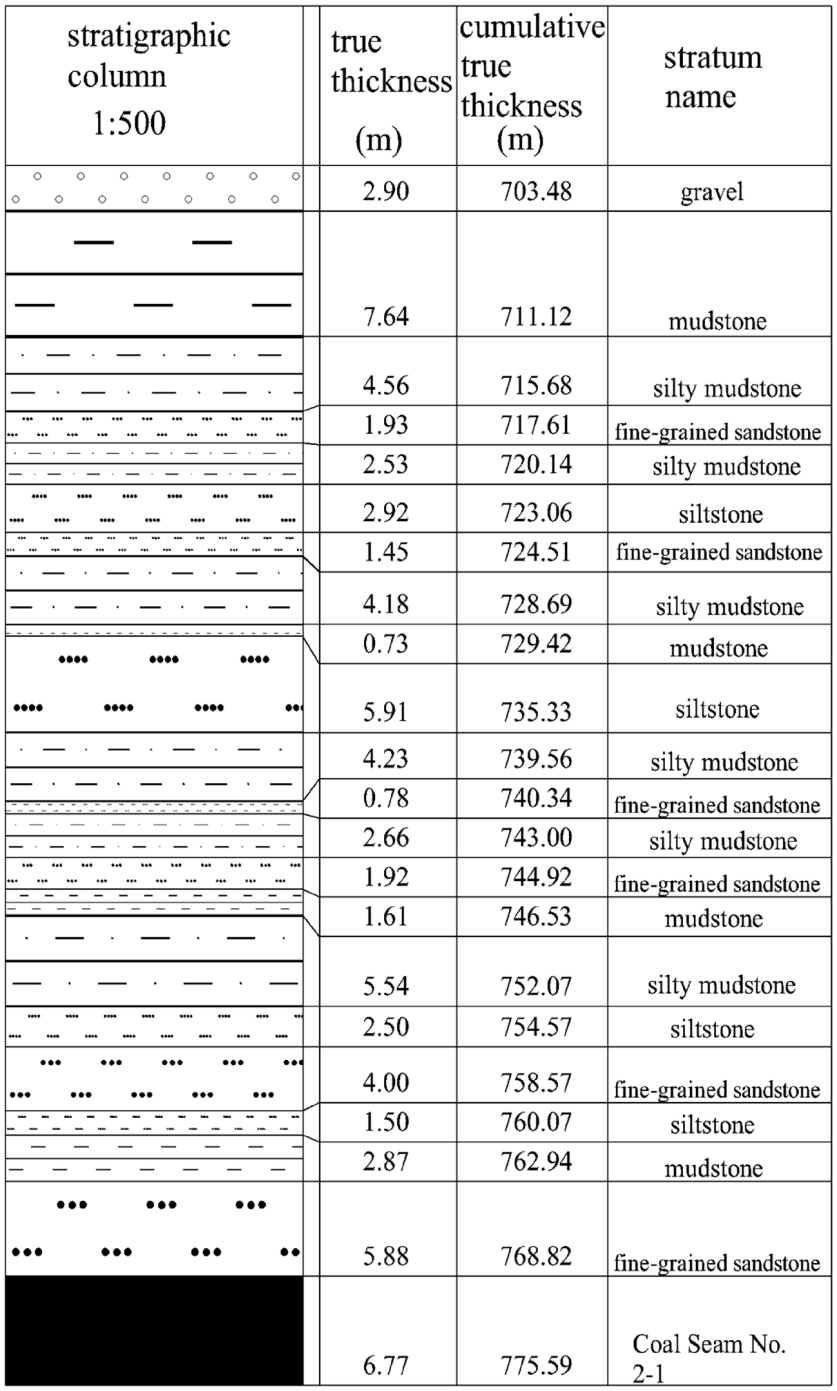
12,801 borehole columnar section.

The main mining II1 coal seam of Zhaogu No. 2 Coal Mine is simple in structure, stable in horizon, and the dip angle is 3°–5°. The 14,022 working face is located in the fourth panel of the mine. At present, the upper layer of the fourth panel has been mined and the lower layer is ready to be mined. In order to realize the one-time mining of the residual coal resources of the section coal pillar, it is planned to adopt the mining technology of “section coal pillar fully mechanized caving + lower layer fully mechanized mining”. The section coal pillar is the protective coal pillar of the mining roadway when mining the upper stratified working face. The dip length of the 14,022 working face is 176 m (35 m in the fully mechanized caving area and 131 m in the fully mechanized mining area), the strike length is 1618 m, the recoverable length of the coal pillar area is 1330 m, and the coal thickness of the lower stratified section of the working face is 1.3–3.5 m. The coal pillar section is 5.1–6.4 m thick, and the plane section of the working face is shown in Fig. 1. The coal seam is dominated by lump coal, and the endogenous fractures are developed, including 1–2 layers of dirt band. The lithology distribution of 12,801 borehole near the working face is shown in Fig. 2.

The upper stratified working face in the working face area is 14,011, 14,021 and 14,011 working face. The end of mining is in December 2013, February 2017 and May 2019, respectively. The roof rust is complete by laying plastic mesh and injecting water into the old pond. The floor aquifer of the working face has been reinforced and transformed by floor grouting, and the aquifer has been transformed into aquifuge or weak water-rich aquifer. The water volume of all roof exploration boreholes is less than 0.5 m3 / h after drainage, and the roof sandstone aquifer is weakly water-rich as a whole. The bedrock is 50.5–94 m thick, and there is a clay layer with a thickness of more than 10 m in the upper part of the bedrock. According to the field measurement of coal seam gas content method and borehole gas leakage method, the effective protection range of 14,022 working face crossing coal pillar (width 35 m) in its adjacent section is determined.

## Mechanical structure model

### Numerical simulation study

**Figure 3:**
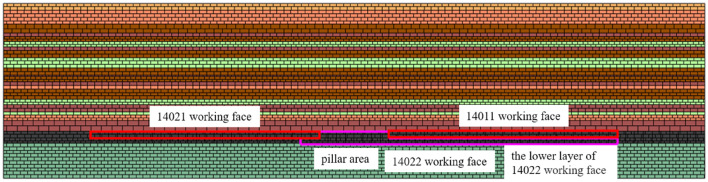
Numerical simulation model.

In order to preliminarily clarify the overlying rock state of the coal pillar area after the mining of the upper layered working face, based on the production geological conditions of the 14,022 working face, the UDEC model was established according to the 12,801 borehole column, and the upper layered working faces on both sides of the coal pillar area and some areas of the 14,022 working face were excavated, shown in Fig. 3. In this simulation, Mohr–Coulomb elastic–plastic model is selected for coal and rock mass, and surface contact Coulomb slip model is selected for joint material model, so as to realize the research on overburden structure such as coal excavation, overburden deformation and stress evolution. The numerical model length (*x*) × height (*y*) = 400 m × 100 m, in which the upper layered excavation areas on both sides of the coal pillar of 14,022 working face are 130 m respectively, and the width of the coal pillar area is 40 m. According to the buried depth, the vertical stress of 14 MPa is applied to the upper part of the model, the lateral pressure coefficient of the model boundary is 1.1, the horizontal and vertical stress gradient is 0.026 MPa, and the fixed boundary constraints are applied to the bottom and both sides of the model. The formation and evolution law of overlying rock structure in stope is simulated.

**Figure 4:**
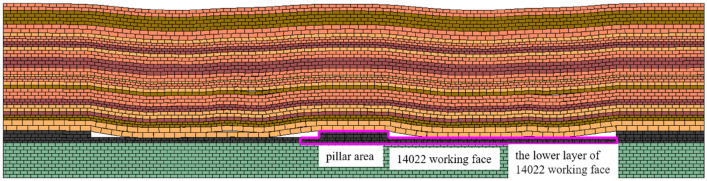
Model after excavation of working face on both sides of coal pillar.

After the mining of the upper layered working face on both sides, the overlying strata fall and the arch structure is presented above the coal pillar, as shown in Fig. 4. The overlying strata in the middle of the upper layered working face are collapsed and compacted, and the upper strata in the coal pillar area are overlapped with the overlying strata on both sides to form an arch structure.

**Figure 5:**
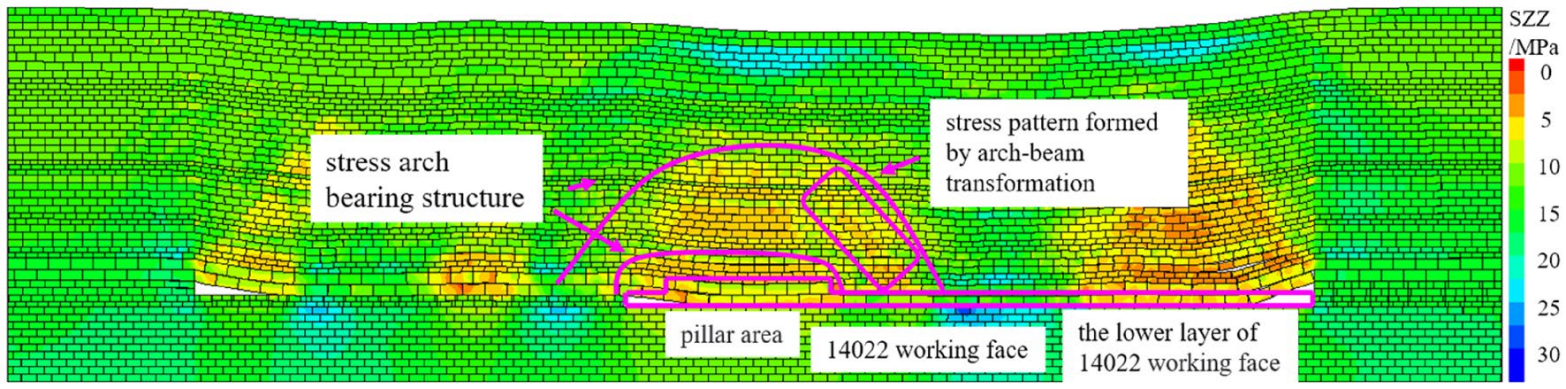
Vertical stress nephogram after mining in pillar area.

After the completion of the upper layer excavation, the 14,022 collaborative mining face was excavated, including the left lower layer part of the coal pillar, the whole coal pillar area and the right lower layer area. The vertical stress cloud diagram of the coal pillar area after mining is shown in Fig. 5. According to the stress distribution, after the excavation of the working face, the direct roof of the coal pillar area is relieved, which corresponds to the collapse of the direct roof overburden. There is a thick hard rock layer above the immediate roof, and the vertical stress increases in this layer, which reflects the pressure-bearing effect of the thick hard rock layer, and the shape of the stress-increasing area is approximately arched. The overburden rock forms a larger range of stress arch bearing structure at a higher level and there is obvious stress concentration at the arch foot. A typical beam-type fracture stress relief zone formed by typical beam-type fracture is presented on the outside of the coal pillar area, that is, the arch-beam conversion of the fracture form occurs at this position.

### Derivation of mechanical structure model

**Figure 6:**
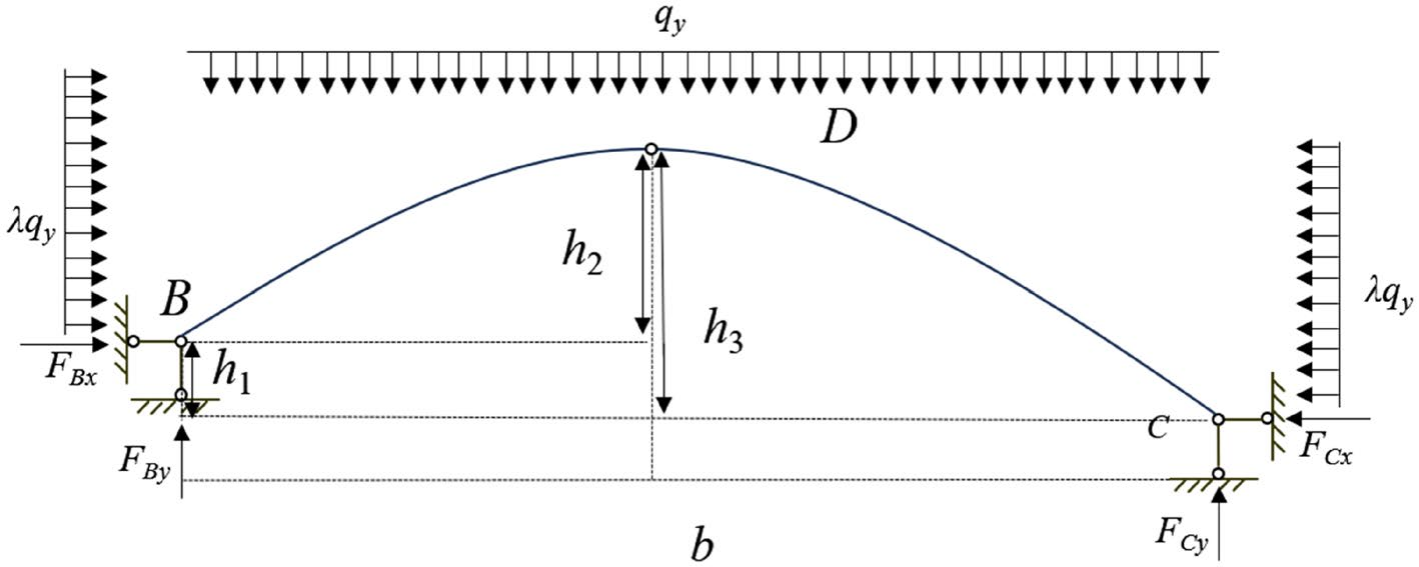
Schematic diagram of hinged stress arch.

According to the results of numerical simulation, it can be seen that there is a stress arch bearing structure above the section coal pillar. At the same time, due to the different mining height and mining influence between the section coal pillar and the lower layer mining area, and the left side of the coal pillar is not affected by the coal seam, the stress arch is simplified into a three-hinge arch structure^29–32^. The middle hinge point of the three-hinge arch is higher than the connection line of the arch foot, forming an inclined three-hinge arch, as shown in Fig. 6. The self-weight of the overburden rock at the upper part of the arch is assumed to be uniformly distributed load *q*, and its lateral abutment pressure is *λq*, where *h*_1_ is the horizontal height difference between the arch foot B and the arch foot C, *h*_2_ is the height difference between the vault and the arch foot B, *h*_3_ is the height difference between the vault and the arch foot C, F_*Bx*_, F_*By*_, F_*Cx*_, F_*Cy*_ are all support reactions.

Assuming the three-hinged arch to be in a stable state with no bending moments, the support reactions can be determined by equilibrium of forces. Analyzing the system as a whole and taking moments about support B yields:

1 $$\Sigma M_{B} = 0$$

2 $$F_{Cx} \cdot h_{3} + q_{y} \cdot b \cdot \frac{b}{2} + \lambda q_{y} \cdot h_{2} \cdot \frac{{h_{2} }}{2} = \lambda q_{y} \cdot h_{3} \cdot \frac{{h_{3} }}{2} + F_{Cy} \cdot b$$

For the isolated segment CD, taking moments about the hinge point D, we have:

3 $$\Sigma M_{D} = 0$$

4 $$F_{Cx} \cdot h_{3} + q_{y} \cdot \frac{b}{2} \cdot \frac{b}{4} + \lambda q_{y} \cdot h_{3} \cdot \frac{{h_{3} }}{2} = F_{Cy} \cdot \frac{b}{2}$$

By combining the above Eqs. (2) and (4), we obtain:

5 $$F_{Cx} = \frac{{q_{y} \cdot b^{2} }}{{4h_{3} }} + \frac{{\lambda q_{y} \cdot h_{2}^{2} }}{{2h_{3} }} - \frac{3}{2}\lambda q_{y} \cdot h_{3}$$

6 $$F_{Cy} = \frac{{3 \cdot q_{y} \cdot b}}{{4h_{3} }} + \frac{{\lambda q_{y} \cdot h_{3}^{2} }}{b} - \frac{2}{b}\lambda q_{y} \cdot h_{3}^{2}$$

From the overall analysis, it can be known:

7 $$F_{By} = \frac{{q_{y} \cdot b}}{4} - \frac{{\lambda q_{y} \cdot h_{2}^{2} }}{b} + \frac{2}{b}\lambda q_{y} \cdot h_{3}^{2}$$

8 $$F_{Bx} = \frac{{q_{y} \cdot b^{2} + 2\lambda q_{y} \cdot h_{2}^{2} }}{{4h_{3} }} + \lambda q_{y} \cdot h_{3}^{2} - \frac{3}{2}\lambda q_{y} \cdot h_{3} - \lambda q_{y} \cdot h_{2}$$

**Figure 7:**
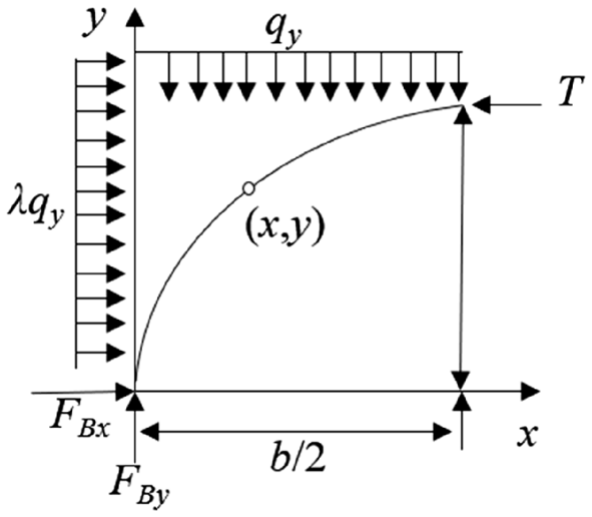
Hinge stress arch mechanics model of overburden in stope.

For any point on the axis of the arch, its bending moment is zero. The rational axis line of the inclined three-hinged arch is as shown in Fig. 7:

Therefore, the rational axis line of the arch is as follows:

9 $$\Sigma M = 0$$

10 $$F_{By} \cdot x = q_{y} \cdot x \cdot \frac{x}{2} + \lambda q_{y} \cdot y \cdot \frac{y}{2} + F_{Bx} \cdot y$$

By substituting Eqs. (7) and (8) into Eq. (10), the axial equation of the inclined three-hinged arch can be obtained:

11 $$x + \lambda y^{2} + \left( {\frac{{2\lambda h_{2}^{2} - 4\lambda h_{3}^{2} }}{b} - \frac{b}{2}} \right)x + \left( {\frac{{b^{2} + 2\lambda h_{2}^{2} }}{{2h_{3} }} - \lambda h_{3} - 2\lambda h_{2} } \right)y = 0$$

**Figure 8:**
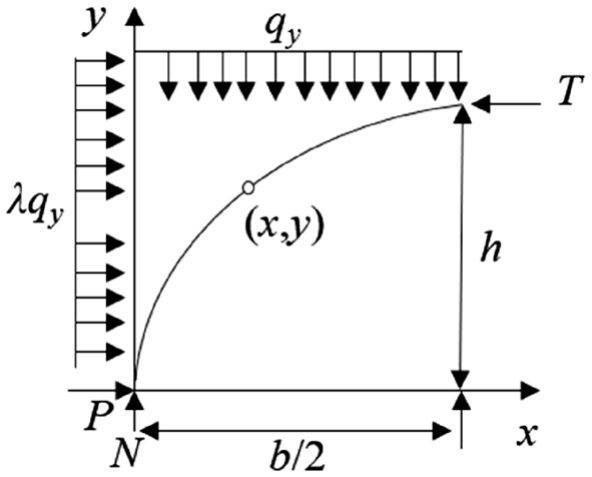
Mechanical model of three-hinged arch.

Equation (11) represents the general form of an ellipse equation, indicating that under similar geological conditions, the minor axis of the inclined three-hinged arch will be significantly smaller than that of a normal three-hinged arch. Therefore, considering the equation of the arch axis line of the three-hinged arch, it is assumed that there is a horizontal tangential support force *T* on the right side of the arch crown towards the left, and horizontal reaction force *P* and vertical reaction force *N* acting at the base of the arch, as shown in Fig. 8.

Therefore, the axial equation of this three-hinged arch is as follows:

12 $$x^{2} + \lambda y^{2} - bx + \frac{1}{h}\left( {\frac{{b^{2} }}{4} - \lambda h^{2} } \right)y = 0$$

Due to the influence of mining activities on the rock layers near the stress arch, the lateral pressure coefficient *λ* is less than 1. Therefore, the lengths of the major and minor axes, 2*a*′ and 2*b*′, respectively, are:

13 $$2a^{\prime} = \frac{{b^{2} }}{4h} + h$$

14 $$2b^{\prime} = \frac{{b^{2} }}{4h\sqrt \lambda } + h\sqrt \lambda$$

It can be seen from the above that after the coal pillar area is mined, the overlying strata collapse and form a stress arch bearing structure at a higher level. The shape of the arch is related to the mining width and the properties of the overlying strata. When the coal pillar area of the cooperative mining face is mined, the stress arch bearing structure formed above can protect the lower mining area and limit the expansion of the overburden failure range.

### Characteristics of mechanical structure model

**Figure 9:**
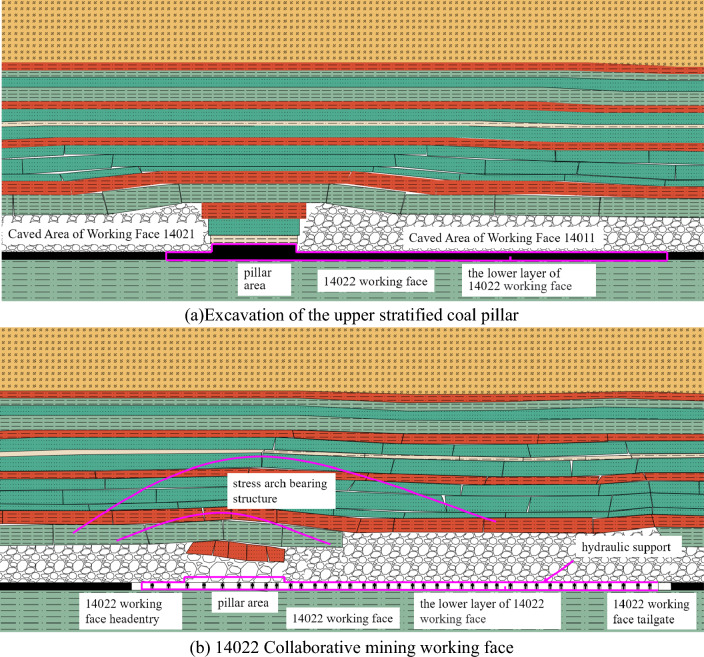
Schematic diagram of hinged stress arch. (**a**) Excavation of the upper stratified coal pillar. (**b**) 14,022 Collaborative mining working face.

Combined with the numerical simulation results, it can be seen that after the recovery of the collaborative mining face, the overlying strata form a stress arch bearing structure, as shown in Fig. 9.

After the upper layered working face on both sides of the section coal pillar is mined, the roof strata fall and fill the goaf under the action of self-weight. The overlying strata on both sides of the section coal pillar are less affected by mining than the central region. The rock layer is broken and compacted gradually in the central region of the upper stratified working face, while the arch structure is formed on both sides of the section coal pillar, as shown in Fig. 9a. After the mining of the working face, combined with Fig. 9b and the numerical simulation results, it can be found that after the mining of the cooperative mining working face, the coal pillar area is different from the lower layered area in mining height and mining influence, and the stress arch bearing structure is formed above the section coal pillar. The stress arch bearing structure above the section coal pillar of the working face mainly has the following characteristics : ① After the mining of the working face on both sides of the section coal pillar, the arch structure is formed above the section coal pillar. When the cooperative mining is carried out, the stress bearing arch structure is formed in the inclined direction of the working face coal pillar area. ② Multiple stress arches can be formed in the dip direction of the working face, and the symmetry of the stress arch in the overlying strata is continuously destroyed and re-formed. ③ Due to the existence of key strata in overlying strata, the upward development of stress arch is also mainly controlled by the combination of rock strata composed of key strata.

## Working face equipment selection

### Hydraulic support in coal pillar area

Hydraulic support is very important to ensure the safe and efficient mining of working face. Determining the reasonable working resistance of hydraulic support is the key factor to ensure the safety of mining, improve the efficiency of mining and prolong the service life of equipment. Through the above mechanical model calculation results combined with the key layer related theory, the working resistance of the hydraulic support in the coal pillar area is determined.

Equation (14) provides the formula for calculating the minor axis of the stress arch below the rational arch axis. To ensure mining safety, considering the maximum height of overburden failure, that is when the semi-minor axis *b*′ equals *h*, the height of the stress arch crown at this time is:

15 $$h = \frac{b}{2}\sqrt {\frac{1}{2\sqrt \lambda - \lambda }}$$

where the span *b* of the stress arch is:

16 $$b = l + 2a = l + 2n\tan \theta$$

where *a* represents the maximum unsupported span of the rock blocks above the height range of failure, and *n* is the height of the caved zone after the recovery of the upper stratification working face. After the recovery of the upper stratification area, the maximum overburden failure height *L* caused by the recovery of the coal pillar area in the collaborative mining working face is:

17 $$L = h + n$$

According to the geological conditions of Zhaogu No. 2 Mine, the empirical formula for calculating the caving zone of medium-hard rock strata in China is selected, and *n* is:

18 $$n = \frac{{100m_{1} }}{{4.7m_{1} + 19}} \pm 2.2$$

where *m*_1_ represents the height of the coal seam in the upper stratification.

By combining the above Eqs. (15)–(18), the maximum overburden failure height *L* due to the recovery of the coal pillar area can be determined as:

19 $$L = \frac{{l + 2\left( {\frac{{100m_{1} }}{{4.7m_{1} + 19}} + 2.2} \right)\tan \theta }}{2}\sqrt {\frac{1}{2\sqrt \lambda - \lambda }} + \frac{{100m_{1} }}{{4.7m_{1} + 19}} + 2.2$$

Combined with the relevant parameters of the coal pillar area of the 14,022 collaborative mining face: the width of the coal pillar area *l* = 35 m, the thickness of the upper layered coal seam *m*_1_ = 3.77 m, the overlying strata caving residual angle *θ* = 15°, the lateral pressure coefficient *λ* = 0.8, substituted into the formula (19) to calculate the maximum failure height of the overlying strata in the coal pillar area* L* = 33.32 m.

From the above 12,801 borehole histogram, it can be seen that the thick and hard siltstone of 5.91 m is 33.49 m from the roof of the coal seam, which is similar to the failure height of the overlying strata in the coal pillar area calculated by the mechanical model, indicating that the thick and hard key stratum has a limiting effect on the upward development of the stress arch, and also proves that the structural model is reasonable.

According to the above theory, when the coal pillar area is mined, the load* Q* of the hydraulic support at the section coal pillar position is :

20 $$Q = L \cdot \gamma \cdot z \cdot (k + n \cdot \tan \theta )$$

Among them, *γ* is bulk density, taking 25 kN/m^3^; *z* is the center distance of the hydraulic support, taking 1.5 m;* k* is the hydraulic support control top distance, take 4.1 m. According to the above values, the working resistance of the hydraulic support can be calculated, and the load that the hydraulic support needs to bear is 9301 kN. The working resistance of the hydraulic support should not be less than 9301 kN. According to the actual situation of the existing hydraulic support in Zhaogu No. 2 Mine, the hydraulic support model of the working face is ZF10000/22/32D.

### Hydraulic support in lower layered area

Zhaogu No. 2 Mine uses a large number of layered mining techniques in the mining of the original working face. Therefore, based on the engineering analogy method, the working resistance of the hydraulic support of the 14,022 collaborative mining face is determined. The object of analogy between the working resistance and support strength parameters of the hydraulic support in the fully mechanized mining area under the 14,022 working face is the 11,012 working face of Zhaogu No. 2 Mine. The lower layer of 14,022 working face and 11,012 working face are different lower layer working faces in the same mine, and their stratum distribution, coal seam occurrence, coal seam roof and floor are similar. The rated working resistance of the hydraulic support currently selected for the 11,012 working face is 9000 kN. In order to maximize the economic and technical effects, the same hydraulic support as the 11,012 working face can be used for the lower layer of the 14,022 working face, that is, the lower layer is determined. The type of hydraulic support is ZZ9000/16/32D (intermediate frame).

### Working face equipment configuration

In view of the geological conditions and safety production of the 14,022 collaborative mining face, the matching selection of the three-machine equipment in the working face is determined to improve the production and operation rate of the working face and achieve safe and efficient production.

The 14,022 cooperative mining face adopts the mining technology of 'section coal pillar fully mechanized caving + lower layered fully mechanized mining', and the goaf is managed by all caving method. The MG300/720-AWD3 shearer is used to cut coal in two directions, and the triangular coal is cut at the end.

The 14,022 front scraper is SGZ764/630. When setting the rear scraper conveyor, a single motor is selected and located at the head position, and the model is SGZ764/315. The transfer carrier is SZZ764/200; BRW400/31.5 Emulsion Pumping Station.

Among them, the hydraulic support of top coal caving adopts the four-column support shield type low top coal caving hydraulic support, the specific model is: ZF10000/20/32D (intermediate frame 31), ZFG10000/20/32D (transition frame 1), ZFG10000/22/35D (end frame 3).

The four-column support shield hydraulic support is selected for the lower layered hydraulic support. The specific models are ZZ9000/16/32D (82 intermediate frames) and ZZG9000/16/32D (3 end frames).

The two types of hydraulic supports adopt the whole roof beam structure with telescopic device, which has strong bearing capacity and can withstand the impact load caused by rock breaking, and can realize the timely support of the broken roof at the front end of the roof beam. In the process of working face mining, when the coal wall is broken, the guard plate can be opened in time to control the rib spalling.

**Figure 10:**
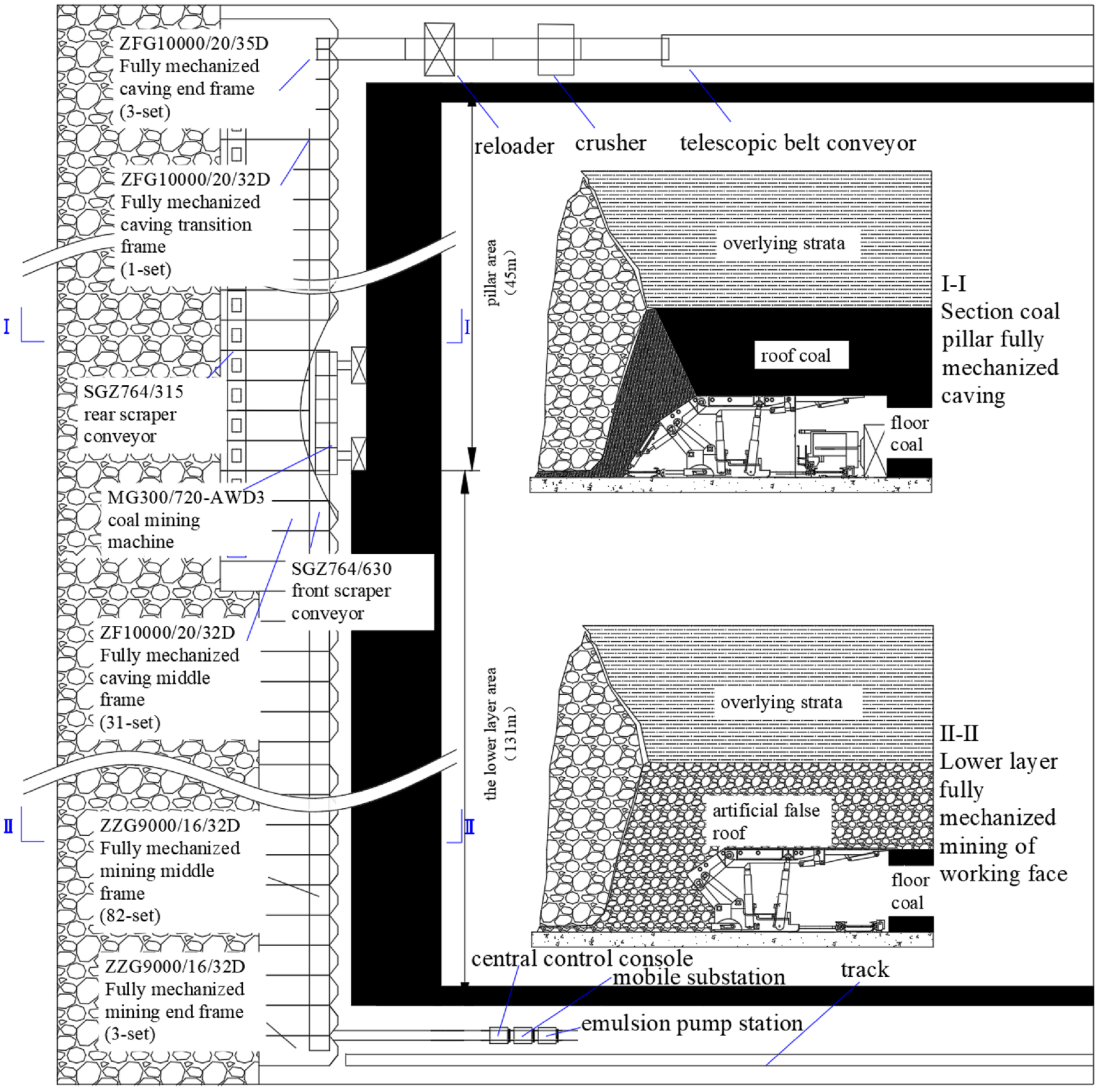
Layout diagram of equipment at the working face.

The specific layout of the working face is shown in Fig. 10. The working face can be divided into two areas, namely the coal pillar area and the lower layer area. The lower layer area adopts the fully mechanized mining technology, using the fully mechanized hydraulic support; the coal pillar area uses the fully mechanized caving process and the fully mechanized caving hydraulic support. In the actual production on site, in order to avoid the gangue channeling of the tail beam after the stubble section and ensure the maintenance space, 3–4 top coal caving hydraulic supports are used in the lower layered section at the junction with the coal pillar section. At the same time, there are 2–3 hydraulic supports in the top coal caving hydraulic support of the coal pillar section near the junction position, so as to prevent the gangue of the artificial false roof in the lower layer section from mixing.

## Adaptability of hydraulic support

Hydraulic support plays an important role in mine pressure behavior, overlying strata movement and its control. Especially for different mining methods in the same working face, the reasonable working resistance of hydraulic support directly affects the control effect of roof. Its adaptability and reliability are the key factors to determine whether the working face can achieve safe and efficient production^33–35^. Based on the selection of hydraulic support and three-machine supporting equipment, the working resistance of hydraulic support, the characteristics of mine pressure and the adaptability of support are analyzed, which is helpful to promote the scientific, efficient and safe production of mine.

### Mine pressure monitoring plan

During the mining process of the working face, the KJ513 mine roof pressure wireless monitoring system was used to monitor the working resistance of the hydraulic support on site. A total of 120 supports were installed in the working face. In order to realize the monitoring of the pressure change of the hydraulic support during the mining process of the working face in the upper and lower ends of the working face, the fully mechanized caving area, the fully mechanized mining area and the fully mechanized caving transition area, a total of 10 measuring points were arranged.

Six consecutive stents were selected for analysis, as shown in Table 1. According to the field situation, the hydraulic support numbering sequence is numbered from the lower crossheading fully mechanized caving area to the upper crossheading fully mechanized mining area^36,37^. The hydraulic support number of the lower crossheading end of the working face is 01, the hydraulic support number of the upper crossheading end is 120, and the installation position of the support stress meter is arranged as shown in the table.

**Table 1:** Layout of strain gauge installation positions on supports.

Location	Support frame number
Fully mechanized caving end frame	10
Fully mechanized caving middle frame	20
Fully mechanized caving transition frame	29
Fully mechanized mining transition frame	31
Fully mechanized middle frame	73
Fully mechanized mining end frame	110

### Analysis of mine pressure monitoring results

#### Characteristics of working resistance of supports

In the process of stable production on site, the monitoring data of each support are shown in the following figure.

**Figure 11:**
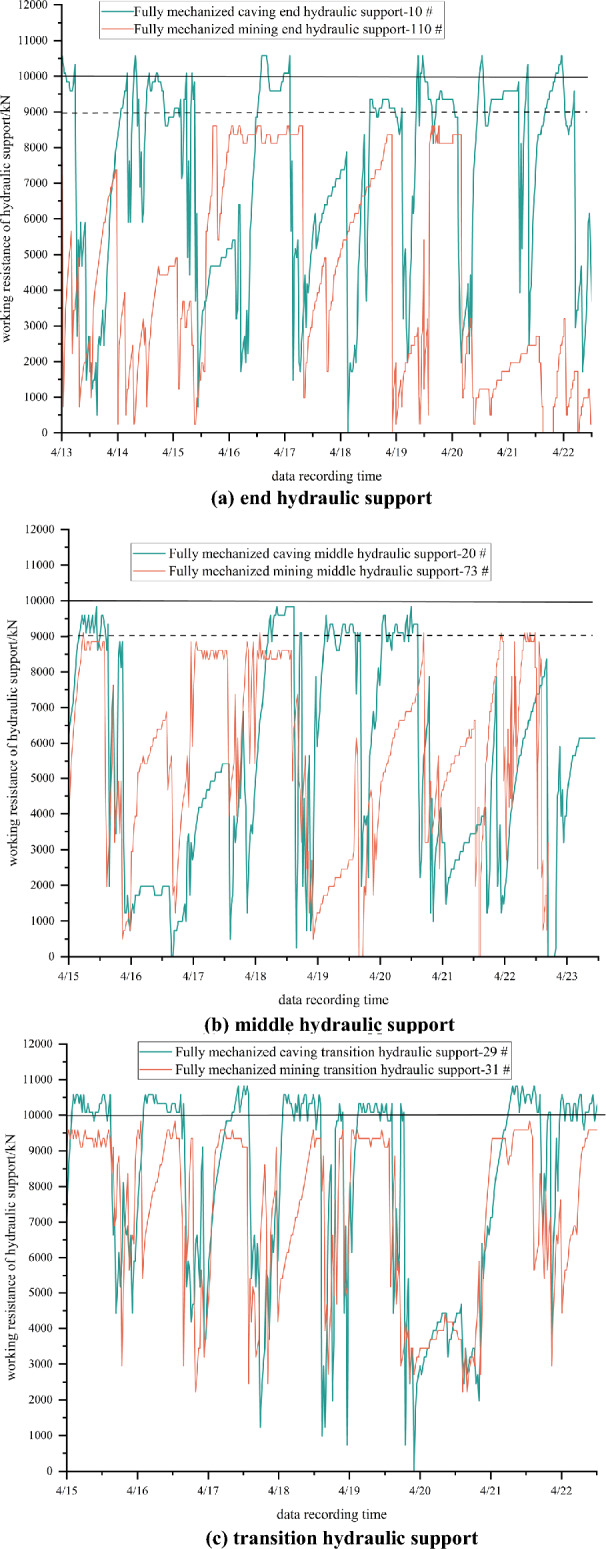
Working resistance of hydraulic supports in different areas. (**a**) End hydraulic support. (**b**) Middle hydraulic support. (**c**) Transition hydraulic support.

Through the analysis of the support pressure data of the end, middle and transition sections of the working face, the working face is in the normal mining stage from April 13, 2023 to April 22, 2023. It can be seen from Fig. 11 that the difference between the fully mechanized caving area and the fully mechanized mining area is obvious. The working resistance of the lower end frame is significantly higher than that of the upper end position. The working resistance of the middle frame in the fully mechanized caving area is less than that in the fully mechanized mining area, but the maximum working resistance of the support in the fully mechanized caving area is greater than that in the fully mechanized mining area. The working resistance of the fully mechanized caving side support in the transition area of the mixed mining face is large. Because the hydraulic support is formed by the technical transformation and upgrading of the old support, the opening of the safety valve is insufficient and not timely, resulting in the support exceeding the rated working resistance. The upper part of the fully mechanized mining side in the transition area is the goaf of the upper layered working face, and its working resistance is significantly reduced compared with the fully mechanized caving side support in the transition area. The above situation reflects that the roof movement at each position of the working face in the tendency direction is not synchronized. The working resistance of the support in the lower layered area is smaller than that in the section coal pillar area. The stress arch bearing structure has a protective effect on the coal pillar area. The stress arch foot is located in the transition position of the fully mechanized caving mining, and the working resistance of the hydraulic support is larger for other positions. The maximum working resistance of the support of the 14,030 large mining height working face in the same panel of the mine is more than 18,000 kN. Through comparison, it can be seen that the pressure of hydraulic support in the coal pillar area of 14,022 collaborative mining face is obviously smaller, which indicates that the stress arch bearing structure has a protective effect on the coal pillar area.

#### Adaptability analysis of hydraulic support

**Figure 12:**
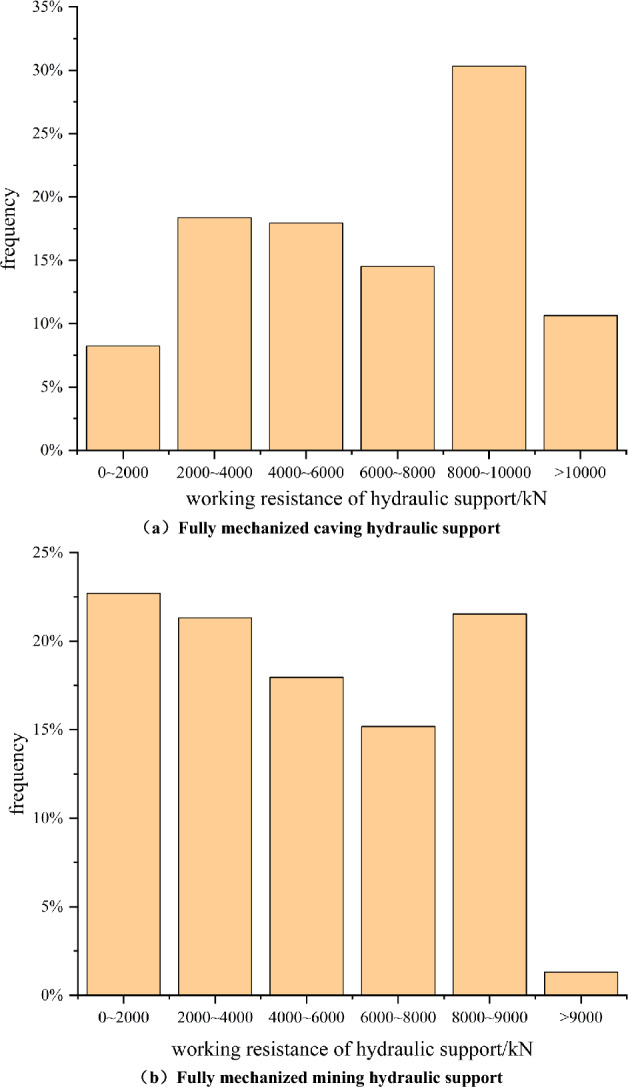
Hydraulic support working resistance histogram. (**a**) Fully mechanized caving hydraulic support. (**b**) Fully mechanized mining hydraulic support.

Figure 12 is the histogram of working resistance frequency of fully mechanized mining and fully mechanized caving support. From the diagram, it can be seen that the distribution of working resistance in the working resistance of the support in the fully mechanized mining area is relatively uniform, and the occurrence of working resistance exceeding the rated working resistance of the support is very rare. The working resistance of the support in the fully mechanized caving area is about 30% in the range of 8000–10,000 kN. Combined with the working resistance of hydraulic support in different areas of Fig. 11, it can be seen that the working resistance of ZF10000/20/32D hydraulic support in the end and middle area of coal pillar area is relatively small, which meets the production requirements. However, the mechanical structure of the transition area is the arch foot of the stress arch structure, which leads to the larger hydraulic support in this position than in other positions in actual production. In order to ensure the safe production and normal operation of the working face, it is necessary to carry out regular inspection and monitoring of the hydraulic support.

In the selection of hydraulic support, the drilling hole closest to the working face is selected to calculate the working resistance of the support. However, there are still differences in the rock strata in the actual mining, and the working resistance of the hydraulic support in some areas is relatively low. In the future, similar working face mining needs to be combined with the actual situation, and the richness coefficient can be increased when selecting the hydraulic support at the section coal pillar position, so as to ensure the safe mining of the working face.

#### Characteristics of mining pressure manifestation

**Figure 13:**
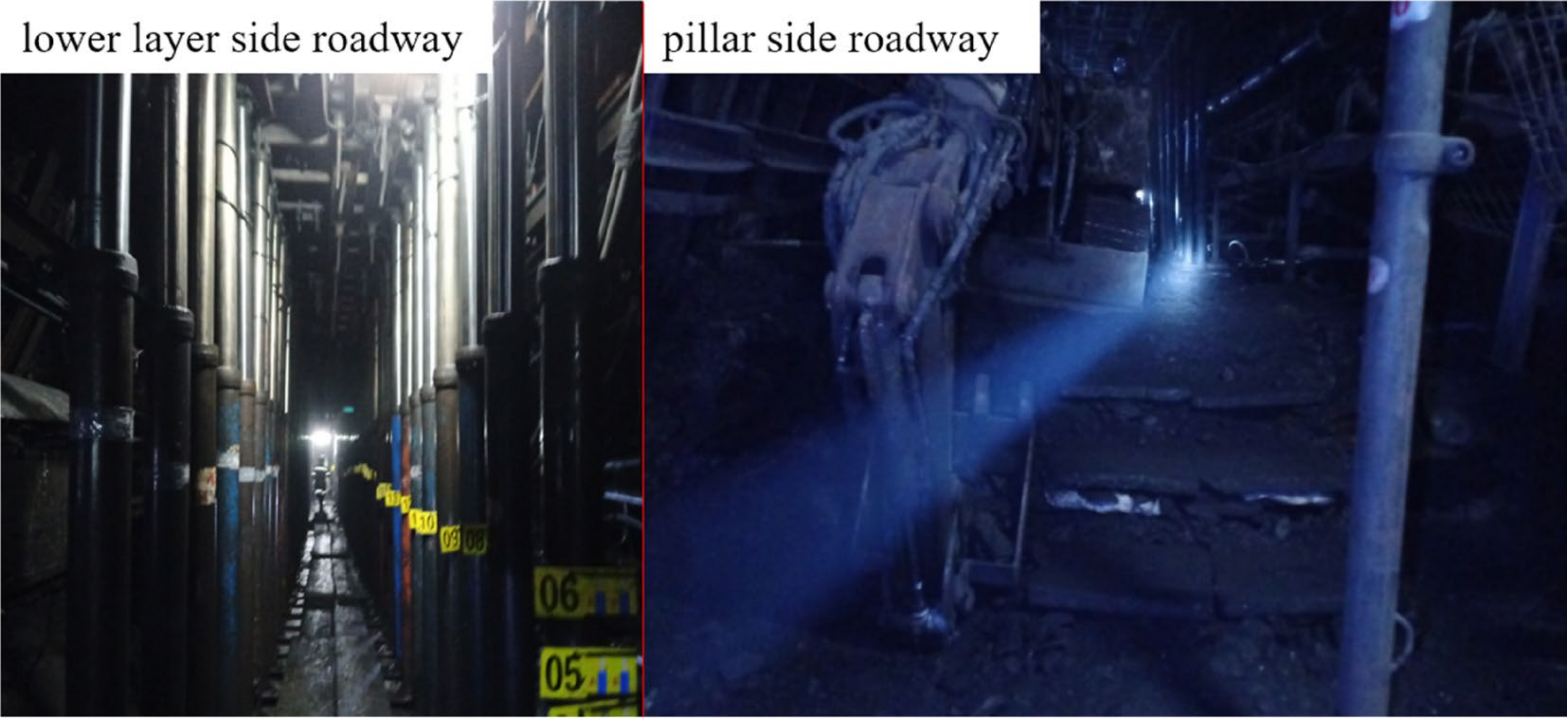
Advanced section of working face roadway.

Through on-site reconnaissance and observation during the mining process of the working face, it is found that the pressure difference between the two roadways in the advanced section of the collaborative mining face is obvious: the pressure of the roadway on the side of the coal pillar is severe, and the integrity of the roadway on the side of the lower layer is better, as shown in Fig. 13. In the field, the advance section of the roadway on the side of the coal pillar is bottomed. The height of the bottom is 1.2–1.3 m, and the height of the roadway after the bottom is about 4.0 m. When the working face is mined to the bottom position, the height of the roadway has converged to 3.4–3.5 m.

**Figure 14:**
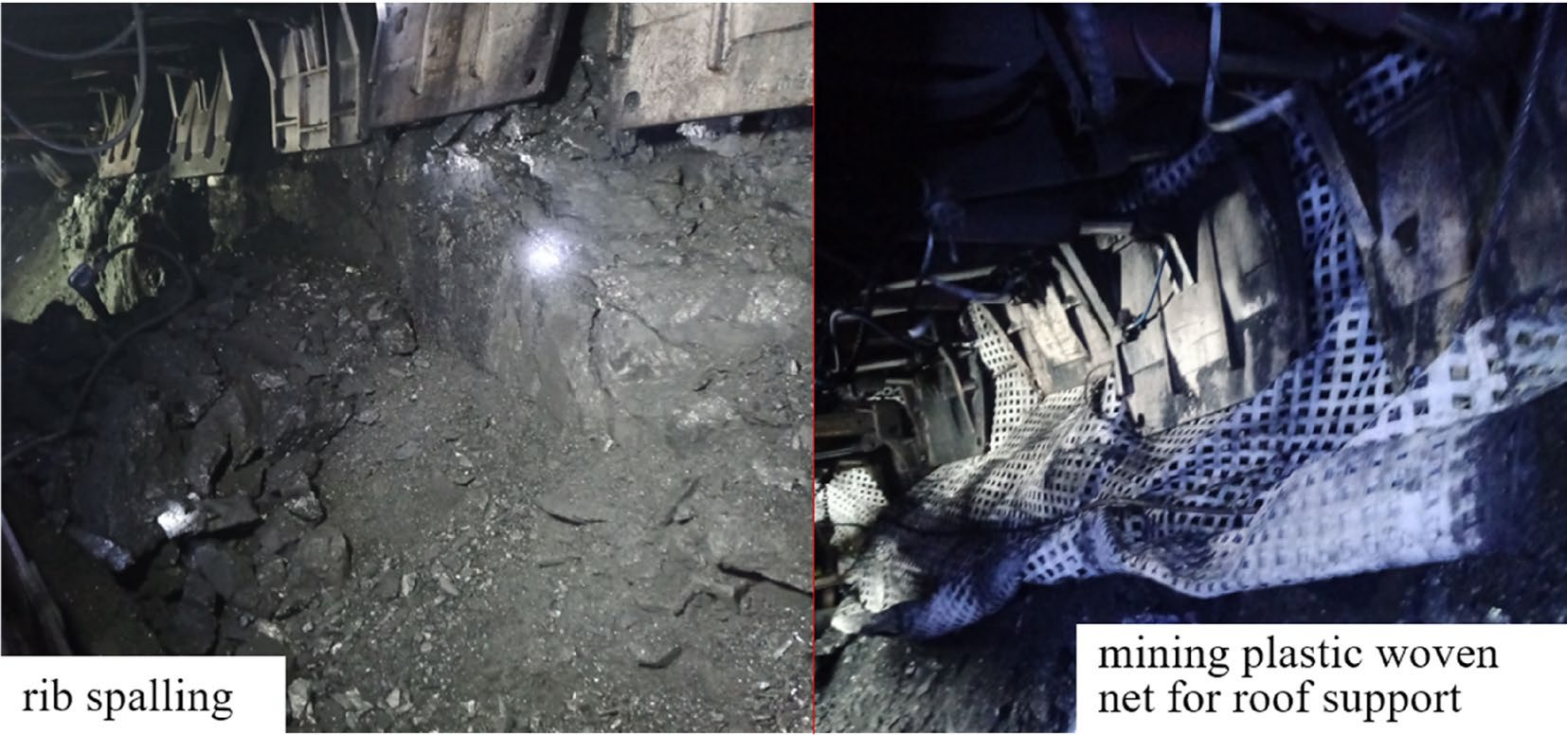
Conditions of the rib in the working face.

When the upper layered working face is mined, the lower coal seam is affected by mining, and the joint fissures of the coal seam are developed. During the mining of the lower layer, the coal wall is prone to spalling due to the influence of the advance abutment pressure. At the same time, due to the existence of the original upper layered working face mining roadway on both sides of the coal pillar area, in order to avoid the occurrence of roof leakage in front of the support, the mining plastic mesh is placed above the support for roof protection, as shown in Fig. 14.

## Conclusions

1. Utilizing the discrete element software, the overburden structure model formed before and after the mining of the working face is established. The model reflects that the stress arch bearing structure can be formed after the mining of the overlying working face and the collaborative mining working face. At the same time, the formation process and characteristics of the stress arch bearing structure are analyzed, and the mechanical model of the inclined three-hinged arch of the overburden stress arch bearing structure is deduced.
2. The calculation formula of working resistance of hydraulic support in section coal pillar area is formed by using the mechanical model of inclined three hinged arch, and the type of hydraulic support in coal pillar area is defined. At the same time, the engineering analogy method is used to select the hydraulic support in the lower layered area. On this basis, the selection of other equipment in the working face, the working face equipment layout are described in detail.
3. Through field measurement data and field investigation, it is shown that ZZ9000/16/32D fully mechanized mining support can meet the requirements of layered roof support under collaborative mining face. ZF10000/20/32D fully mechanized caving support is in the arch foot of stress arch structure in the transition area of fully mechanized caving mining in production, and the working resistance is larger than other positions. The monitoring and inspection should be strengthened in the mining process. In general, these research results can provide reference for the recovery of residual coal in section coal pillar.

## Data Availability

Some or all data, models, or codes generated or used during the study are available from the corresponding author by request.
